# Advancing Sensitivity in Guided-Wave Surface Plasmon Resonance Sensor through Integration of 2D BlueP/MoS_2_ Hybrid Layers

**DOI:** 10.3390/bios14010025

**Published:** 2023-12-31

**Authors:** Xixi Yuan, Leiming Wu, Yuwen Qin

**Affiliations:** 1 College of Electronics and Information Engineering, Shenzhen University, Shenzhen 518060, China; yuanxixi@szu.edu.cn; 2 Key Laboratory of Photonic Technology for Integrated Sensing and Communication, Ministry of Education of China, Guangdong University of Technology, Guangzhou 510006, China; qinyw@gdut.edu.cn

**Keywords:** surface plasmon resonance, sensors, guide-wave SPR, blueP/MoS_2_ hybrid layer

## Abstract

The surface plasmon resonance (SPR) signal, generated from the Kretschmann configuration, has been developed as an effective detection technology in chemical and biological sensors. The sensitivity of SPR signals to changes in the surrounding media makes it a valuable tool, as even a slight variation in refractive index can cause a significant change in SPR signals, such as phase, intensity, and resonance angle. However, the detection of ultralow changes in refractive index, which occur in chemical reactions or biological actions, remains a challenge for conventional SPR sensors due to their limited sensitivity. To overcome this limitation, we theoretically propose a novel guided-wave SPR (GWSPR) configuration coated with a few-layer blue phosphorene (blueP)/MoS_2_ hybrid structure. This configuration aims to enhance the electric field and subsequently achieve a significant improvement in sensitivity. The results of our study demonstrate that the proposed blueP/MoS_2_-based GWSPR sensor exhibits a high sensitivity of 290°/RIU, which represents an impressive enhancement of approximately 82.4% compared to the conventional Au-based SPR sensor. This advancement addresses the challenge of detecting ultralow changes in refractive index and offers significant potential for enhancing the performance of chemical and biological sensors.

## 1. Introduction

Surface plasmon resonance (SPR) is an oscillation of free charge at the interface between metal and dielectric [1,2,3]. When incident light undergoes total reflection at the interface between dielectric and metal, it generates an evanescent wave that excites the free electrons on the metal surface, forming the surface plasma wave (SPW). When the wave vector of the SPW matches the wave vector of incident light in the horizontal direction, the SPR effect is excited [4,5]. Meanwhile, most of the energy of the incident light is transferred to support the resonance effect, causing the reflectance curve to sharply drop at the resonance angle, forming the resonance dip. This dip, known as the SPR signal, is sensitive to changes in the refractive index of the surrounding media. The SPR signal is now widely used to detect biological interactions and chemical reactions, such as in medical diagnosis [6,7,8], drug discovery [9,10,11], food safety [12,13,14], and environmental monitoring [15,16,17]. Every change in the refractive index of the sensing medium results in a noticeable alteration in the SPR signal, reflected in the movement of the resonance angle in the reflectance curve. By analyzing these altered SPR signals, we can monitor the process of biological interaction or chemical reaction in real time.

However, conventional SPR sensors still face significant challenges in detecting biological or chemical information, such as small molecular compounds, ultra-low concentrations of analytes, and weak interactions. The conventional Au-based SPR sensor lacks the sensitivity required to monitor ultra-weak changes in the sensing medium, necessitating a new SPR configuration with high sensitivity. In an effort to enhance the detection sensitivity of SPR sensors, Abdulhalim et al. [18,19] proposed a novel GWSPR configuration in which a high refractive index dielectric layer is coated on the surface of the Au film. The result of this study found that the GWSPR configuration greatly enhanced the electric field intensity at the sensing interface, thereby improving the sensitivity of the SPR sensor. In addition to the GWSPR configuration, 2D materials have garnered widespread attention in recent years for their potential to improve sensitivity in SPR sensors. Graphene, as the first discovered 2D material, has been shown to possess excellent biocompatibility [20]. The adsorption of biomolecules to graphene is stronger and more stable than that to the Au surface [21] due to π-stacking interactions [22,23]. Because of these advantages, graphene is widely used in biosensors, leading to improved biocompatibility and sensitivity. Zeng et al. [24] reported an ultrasensitive SPR sensor achieved by coating a few-layer graphene on the surface of a conventional SPR sensor. The result demonstrated that the electric field at the sensing interface can be greatly enhanced by coating monolayer graphene on the Au surface, resulting in a sensitivity improvement of about three orders of magnitude.

In addition to graphene, other emerging 2D materials, such as anti-monene [25,26], MoS_2_ [27,28], black phosphorus (BP) [29,30], MXene [31,32], hold promise as sensing materials for enhancing the sensitivity of traditional SPR sensors. When these 2D materials are deposited onto the surface of metallic films, a robust coupling effect is generated at the metal/graphene interface due to effective charge transfer, resulting in a substantial enhancement of the electric field at the sensing interface [33,34,35,36]. Similarly, the blueP/MoS_2_ hybrid layer, another novel 2D material, demonstrates outstanding sensing capabilities. Certain dislocation of constituent phosphorus atoms can convert the puckered structure of black phosphorene into a more symmetric buckled structure, which is referred to as blue phosphorene (BlueP) [37,38]. BlueP and MoS_2_ monolayers share the same hexagonal crystal structure, making it possible to construct appropriate BlueP/MoS_2_ van der Waals heterostructures. With a lattice constant of 3.268 Å and a manageable lattice mismatch of 3.18% relative to MoS_2_, the creation of a heterostructure can be easily achieved by stacking blueP on top of MoS_2_ [39]. The single-layer BlueP and MoS_2_ both have a large specific surface area, which can form a wrinkled surface, providing more space for sensing and detection [40,41]. In this investigation, we explore the application of a GWSPR sensor incorporating a blueP/MoS_2_ hybrid layer to enhance the electric field and sensitivity.

## 2. Design Consideration and Methods

**Figure 1:**
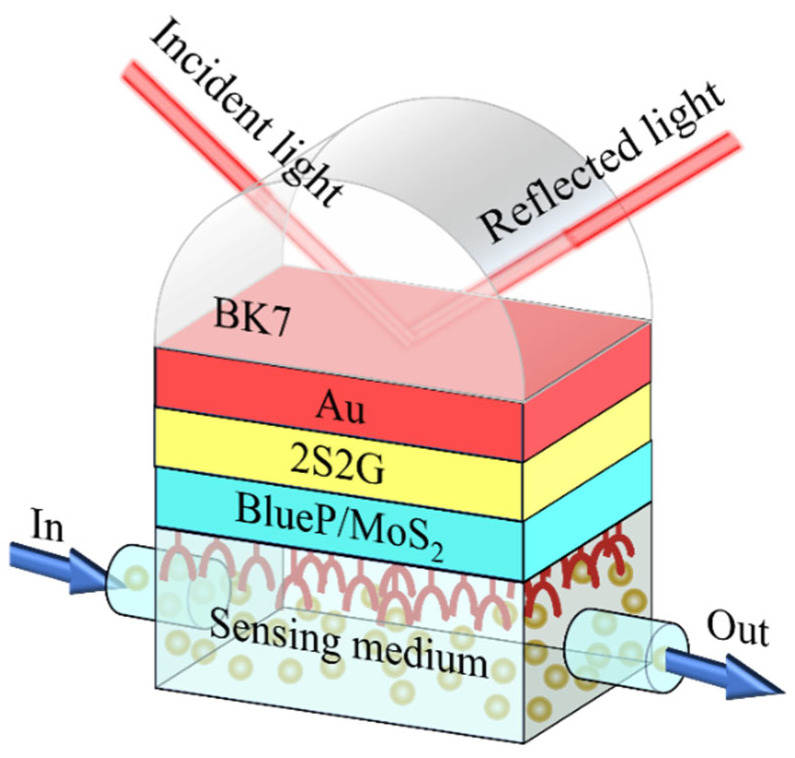
Schematic diagram of the proposed GWSPR biosensor using 2S2G and blueP/MoS_2_ to enhance the sensitivity.

This investigation introduces a novel GWSPR sensor based on blueP/MoS_2_ (Figure 1) to detect slight variations in the surrounding environment’s refractive index. In the proposed GWSPR configuration, a BK7 glass is used as the coupling prism [27,42,43], a common material in SPR sensing technology known for its relatively low refractive index, enabling higher sensitivity [44]. The second layer comprises a thin film of Au, chosen for its ability to efficiently excite surface plasmon polaritons (SPP) and strong antioxidant capacity for stability. The thickness of this layer is 40 nm. The third layer is chalcogenide (2S2G), a low-loss guide-wave layer aimed at enhancing the electric field and sensitivity, commonly used in SPR sensors [45,46,47,48,49]. The thickness of this layer can be optimized during the design process of the sensing structure, and the optimization process is presented in the Section 3. Subsequently, a monolayer of blueP/MoS_2_ hybrid material, with a thickness of 0.75 nm [39,50], is coated on the surface of the 2S2G layer to further improve the sensor’s sensitivity. The final layer is the sensing medium, where chemical reactions or biological effects occur, with a refractive index of 1.33 + ∆*n_s_*, where ∆*n_s_* represents the change in the refractive index of the sensing medium. The refractive index of each layer at λ = 633 nm in the proposed GWSPR configuration can be found in Table 1.

Using the transfer matrix method (TMM), the reflectance (R) curves of the proposed GWSPR sensor can be calculated. The TMM is given as [51,52]:

(1) $$M=\prod_{k=2}^{N-1}M_{k}=\left[\begin{array}{l} M_{11}\;M_{12}\\ M_{21}\;M_{22} \end{array}\right],$$

with

(2) $$M_{k}==\left[\begin{array}{l} \cos\beta_{k}\;\;\;\left(-i\sin\beta_{k}\right)/q_{k}\\ -iq_{k}\sin\beta_{k}\;\;\;\cos\beta_{k} \end{array}\right],$$

where

(3) $$\beta_{k}=\frac{2\pi d_{k}}{\lambda }{\left(\varepsilon_{k}-n_{1}^{2}\sin^{2}\theta \right)}^{1/2},$$

and

(4) $$q_{k}=\frac{{\left(\varepsilon_{k}-n_{1}^{2}\sin^{2}\theta \right)}^{1/2}}{\varepsilon_{k}},$$

where *θ* is the incident angle and *n*_1_ is the refractive index of prism. Then the total reflection coefficient *r_p_* is defined as:

(5) $$r_{p}=\frac{\left(M_{11}+M_{12}q_{N}\right)q_{1}-\left(M_{21}+M_{22}q_{N}\right)}{\left(M_{11}+M_{12}q_{N}\right)q_{1}+\left(M_{21}+M_{22}q_{N}\right)}.$$

And the reflectance (*R_p_*) is written as:

(6) $$R={\left|r_{p}\right|}^{2}.$$

The angle corresponding to the minimum *R* is known as the resonance angle (*θ_res_*), and the sensitivity is defined as [19,44,48]:

(7) $$S=\frac{\Delta \theta_{res}}{\Delta n_{s}},$$

where ∆*θ_res_* represents the change in the resonance angle caused by ∆*n_s_*.

Additionally, the sensitivity enhancement (SE) can be expressed as:

(8) $$SE=\frac{S_{L}-S_{0}}{S_{0}},$$

where *S_L_* is the sensitivity for the proposed GWSPR sensor when the number of blueP/MoS_2_ hybrid layers is *L*, and *S*_0_ is the sensitivity of a conventional Au-based SPR sensor. Furthermore, the figure of merit (FOM) is another important performance indicator and can be defined as [47]:

(9) $$FOM=\frac{S}{FWHM}$$

where *FWHM* is the full width at half maximum.

## 3. Results and Discussions

**Figure 2:**
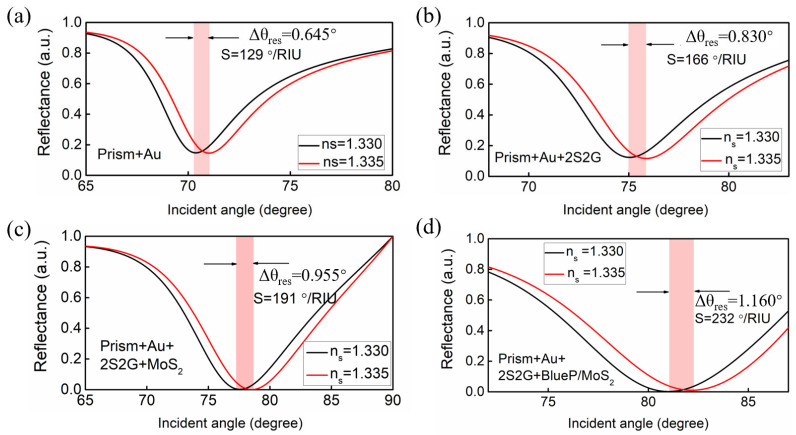
The reflectance curves as a function of incident angle for (**a**) the conventional prism + Au based SPR sensor, (**b**) prism + Au + 2S2G based guide-wave SPR sensor, (**c**) prism + Au + 2S2G + MoS_2_ based guide-wave SPR sensor, and (**d**) the proposed GWSPR sensor based on prism + Au + 2S2G + blueP/MoS_2_.

The excitation wavelength is 633 nm (He-Ne laser is usually used in practical applications) in the proposed theoretical GWSPR sensor. The BK7 prism, characterized by its relatively low refractive index, is employed as a coupling prism in the proposed GWSPR sensor, enabling it to produce greater sensitivity compared to prisms with higher refractive indices [44]. According to Snell’s law, a low refractive index coupling prism can yield a substantial change in the resonance angle, thereby enhancing sensitivity in SPR sensors. Subsequently, a 40 nm thick Au thin film is applied to the surface of the BK7 prism to serve as the metal layer. The phenomenon of SPR can be observed when the TM-polarized light is reflected from the BK7 coupling prism. The resulting reflectance curve, known as the SPR signal, is highly responsive to changes in the refractive index of the sensing medium (*n_s_*), making it widely applicable in biosensing, as well as in chemical and environmental monitoring [53,54,55]. Our results indicate that the SPR detection signal of the Au-based sensor (Figure 2a) progressively intensifies with the incorporation of the 2S2G guide-wave layer (Figure 2b) and the 2D blueP/MoS_2_ layers (Figure 2d).

**Figure 3:**
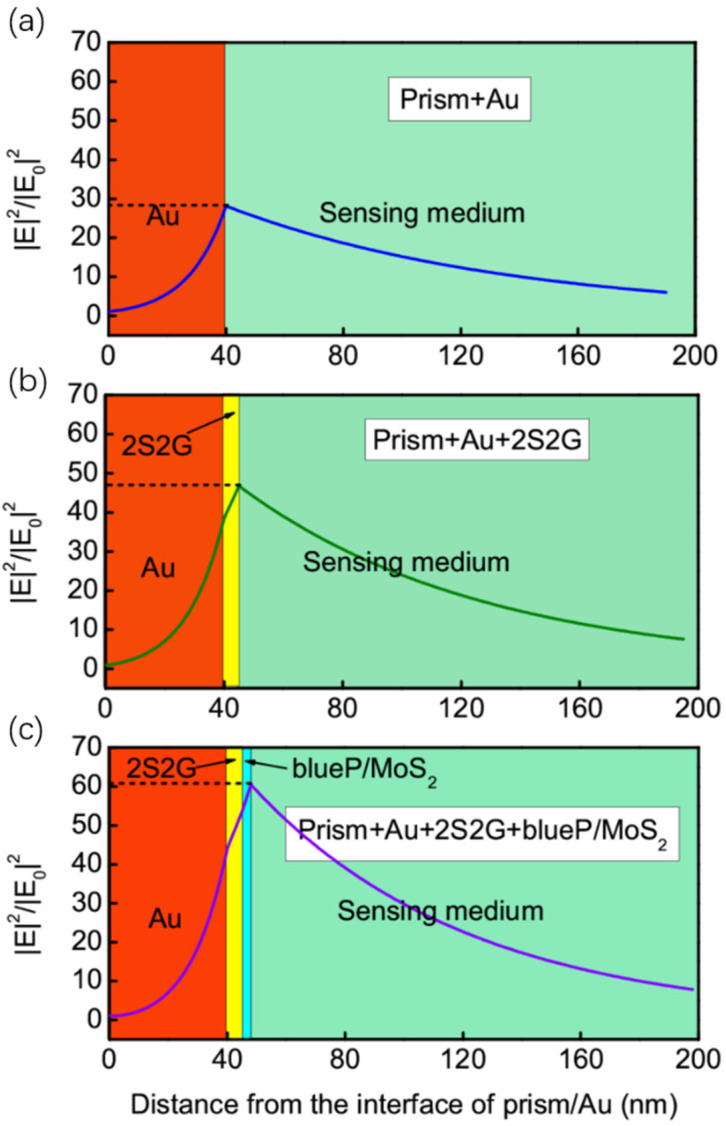
Normalized electric field distributions for the (**a**) prism + Au based SPR sensor, (**b**) prism + Au + 2S2G based SPR sensor, and (**c**) prism + Au + 2S2G + blueP/MoS_2_ based SPR sensor.

When the 2S2G guide-wave layer and 2D blueP/MoS_2_ layer are deposited on metallic thin films, the electric field at the sensing interface can be significantly enhanced (Figure 3). The induced electric field on the surface of Au forms an evanescent wave, which is highly responsive to changes in the refractive index of the surrounding environment. A stronger electric field indicates that higher sensitivity can be achieved, enabling the detection of even slight changes in the *n_s_*. The normalized electric field at the sensing interface for the conventional Au-based SPR sensor is 28.14 (Figure 3a), but this level of enhancement is not robust enough to provide high sensitivity. In order to improve the electric field at the sensing interface, the GWSPR configuration includes the coating of a dielectric layer of 2S2G on the surface of the Au film to act as the guide-wave layer and further enhance the electric field. The results demonstrate that the normalized electric field at the sensing interface increases to 46.78 by covering a 5 nm thick 2S2G layer on the surface of the Au thin film (Figure 3b).

In recent year, 2D materials have been investigated for their potential application in SPR sensors due to their adsorption characteristics [20]. Previous reports [24,29] have indicated that coating few-layer 2D materials on the surface of the Au film can improve the electric field. Herein, we coat 4 layers of blueP/MoS_2_ hybrid 2D material on the surface of the GWSPR sensor to further enhance the electric field at the sensing interface. Upon calculation, the electric field of the 2D blueP/MoS_2_-based GWSPR sensor is found to be improved to 60.31 (Figure 3c), representing a 2.14-fold increase compared to the conventional Au-based SPR sensor.

With the enhancement of the electric field at the sensing interface, the sensitivity has been significantly boosted. The initial sensitivity for the conventional Au-based SPR sensor is 129°/RIU (Figure 2a). However, with the addition of a 2S2G guide-wave layer and a blueP/MoS_2_ hybrid layer, the sensitivity gradually improves as a result of the enhanced electric field. Coating a 5 nm thick 2S2G on the Au surface can improve the sensitivity to 166°/RIU (Figure 2b). By attaching a layer of MoS_2_ (the thickness of monolayer MoS_2_ is 0.65 nm) on top of 2S2G, the sensitivity of the sensor will increase again (Figure 2c). Furthermore, covering the GWSPR sensor with 4 layers of blueP/MoS_2_ hybrid 2D material can achieve a sensitivity as high as 232°/RIU (Figure 2d).

**Figure 4:**
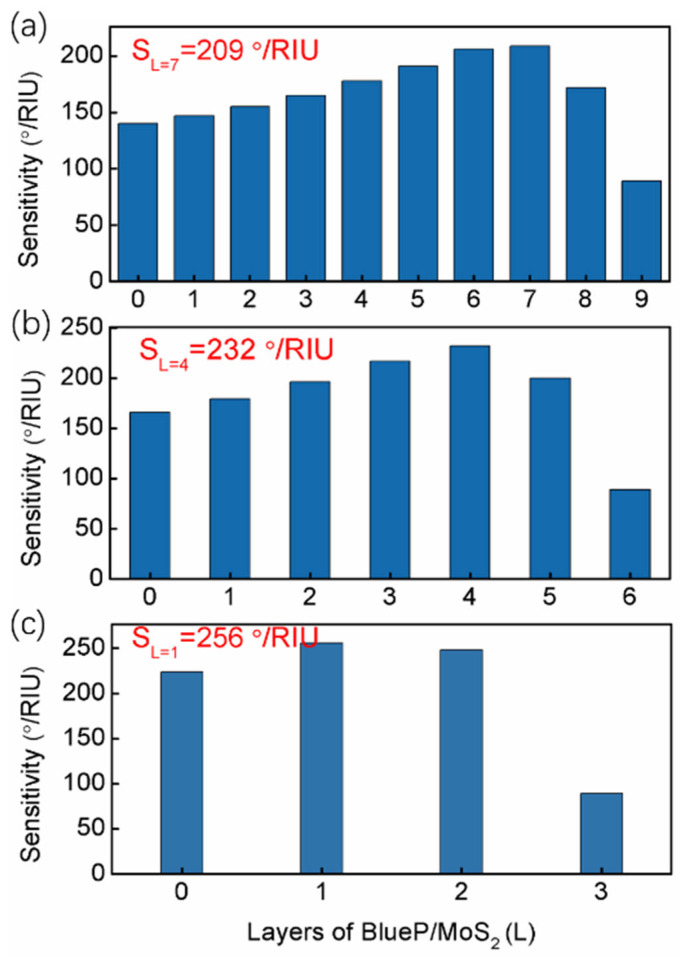
Sensitivity of the proposed GWSPR sensor as a function of layers at different 2S2G thickness, (**a**) d2S2G = 2 nm, (**b**) d2S2G = 5 nm, and (**c**) d2S2G = 8 nm.

To achieve the highest sensitivity, we optimized the thickness of the guide-wave layer and the number of blueP/MoS_2_ layers (L). Setting the 2S2G thickness to 2 nm, the sensitivity for the proposed GWSPR sensor reached its peak value at an optimum of 7 blueP/MoS_2_ layers (S = 209°/RIU) (Figure 4a). Beyond this point, sensitivity began to decline. Likewise, when the 2S2G thicknesses were set at 5 nm (Figure 4b) and 8 nm (Figure 4c), the optimum number of blueP/MoS_2_ layers were 4 (S = 232°/RIU) and 1 (S = 256°/RIU), respectively. For comparison, the optimal configuration for the proposed GWSPR sensor is an 8 nm thick 2S2G coated with a monolayer of blueP/MoS_2_ hybrid material at *n_s_* = 1.33.

**Figure 5:**
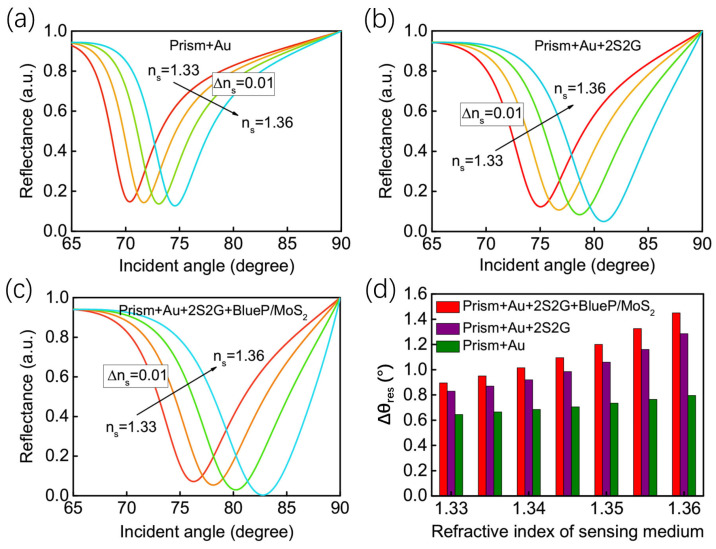
Variation of reflectance curves when the refractive index of sensing medium ranges from 1.33 to 1.36 for the sensors of (**a**) prism + Au, (**b**) prism + Au + 2S2G, and (**c**) the prism + Au + 2S2G + blueP/MoS_2_. (**d**) Comparison of Δ*θ_res_* for three different types of SPR sensors when Δ*n_s_* = 0.005.

The proposed 2S2G-blueP/MoS_2_-based GWSPR sensor can also be utilized to detect other refractive index changes beyond *n_s_* = 1.33. The variation of reflectance curves for the SPR sensors based on prism + Au, prism + Au + 2S2G and prism + Au + 2S2G + blueP/MoS_2_ are shown in Figure 5a–c. The results demonstrate that the proposed 2S2G-blueP/MoS_2_-based GWSPR sensor can obtain the strongest resonance under the same conditions, and the resonance angle will move to a larger value when the refractive index of the sensing medium increases from 1.33 to 1.36. It is worth noting that the proposed sensor is based on angular detection, which has a limitation in angle range, and the angular limitation is 90°. When the *n_s_* > 1.36, the sensitivity will begin to decrease owing to the angular limitation. Therefore, the refractive index detection range of the proposed sensor is 1.33 to 1.36. The change in *n_s_* can cause the shift of resonance angle, and the ∆*θ_res_* is obtained in Figure 5d when the Δ*n_s_* = 0.005. The result indicates that the proposed 2S2G-blueP/MoS_2_-based GWSPR configuration is the most sensitive sensor to generate the largest ∆*θ_res_*.

**Figure 6:**
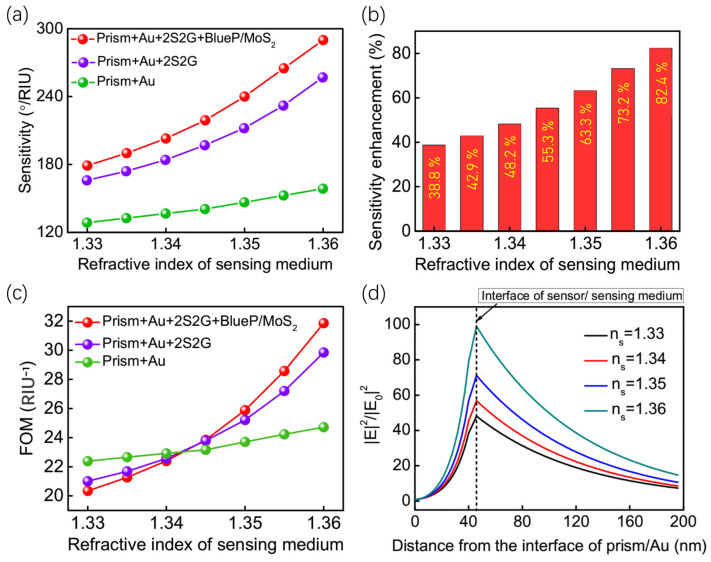
(**a**) Sensitivity as a function of refractive index of sensing medium for different SPR structures: prism + Au, prism + Au + 2S2G, and prism + Au + 2S2G + blueP/MoS_2_; (**b**) Sensitivity enhancement of the proposed GWSPR sensor (prism + Au + 2S2G + blueP/MoS_2_) comparing to the conventional Au-based SPR sensor; (**c**) The FOM values for three different SPR sensors; (**d**) The electric field distribution of the proposed sensor based on prism + Au + 2S2G + blueP/MoS_2_ when the refractive index of sensing medium change from 1.33 to 1.36.

The sensitivity of the proposed 2S2G-blueP/MoS_2_-based GWSPR sensor was calculated and compared with another structure involving prism + Au and prism + Au + 2S2G. This comparison was conducted over a refractive index range of 1.33 to 1.36 (Figure 6a). The results revealed that the 2S2G-blueP/MoS_2_-based GWSPR configuration achieved the highest sensitivity within the refractive index range of (1.33, 1.36) among the different SPR configurations, with the highest sensitivity value recorded at 290°/RIU at *n_s_* = 1.36 (Figure 6b). Additionally, the SE for the proposed GWSPR sensor was calculated in Figure 6b, demonstrating the superiority of the proposed sensor, with the highest SE reaching 84% at *n_s_* = 1.36.

Furthermore, the figure of Merit (FOM) for these three sensor types was calculated in Figure 6c. It was observed that the conventional Au-based SPR sensor had a larger FOM than the other two GWSPR sensors in the *n_s_* range of (1.33, 1.34). However, for *n_s_* > 1.34, the proposed 2S2G-blueP/MoS_2_-based GWSPR sensor outperformed the other two sensors, exhibiting a larger detection range. Moreover, the electric field for the proposed GWSPR sensor was found to be sensitive to changes in *n_s_*. As *n_s_* gradually changed from 1.33 to 1.36, the electric field exhibits a significant change at the sensing interface, and the electric field was enhanced to a higher value with increasing refractive index (Figure 6d). With such exceptional detection performance, the proposed 2S2G-blueP/MoS_2_-based GWSPR sensor demonstrates promising potential for future sensing technology applications.

In Table 2, we have provided a comparison of the results from our proposed work with previous reports. Typically, a 633 nm wavelength light source is used to excite the surface plasmon resonance (SPR) of the sensing configuration. In recent years, there has been a significant focus on utilizing 2D materials to enhance sensitivity. For instance, the use of Au coated with BP/graphene hybrid improved sensitivity to 218 °/RIU at λ = 633 nm [56], while the employment of Au coated with BP/WS_2_ hybrid enhanced sensitivity to 279°/RIU at λ = 633 nm [29]. Furthermore, the use of Ag coated with PtSe_2_/graphene hybrid increased sensitivity to 235°/RIU at λ = 633 nm [57]. Additionally, it is important to note that changing the wavelength of the excited light can also lead to improved sensitivity. For example, Sharma et al. reported a SPR sensor based on Ag/BlueP/MoS_2_ with a sensitivity of 355°/RIU at λ = 662 nm [50]. However, this wavelength is not commonly used in practical applications. Our comparison in Table 2 underscores the high sensitivity of our proposed SPR sensor in relation to these previous reports.

## 4. Conclusions

In summary, we have introduced a novel GWSPR sensor utilizing a 2D blueP/MoS_2_ hybrid layer to enhance the electric field and detection sensitivity. Initially, a 2S2G layer with a high refractive index is applied to the surface of the Au film to serve as a guide-wave layer. The outcome illustrates a significant improvement in sensitivity due to the enhancement of the electric field within the guide-wave layer. Furthermore, the incorporation of the 2D blueP/MoS_2_ hybrid layer is leveraged to further enhance the sensitivity of the proposed GWSPR sensor. Integration of the 2D blueP/MoS_2_ hybrid layers results in a subsequent improvement in the electric field and sensitivity. Consequently, the optimal configuration for the proposed GWSPR sensor is determined to be an 8 nm thick 2S2G layer coated with a monolayer of blueP/MoS_2_ hybrid layer. In addition, future research on SPR sensors based on 2D materials may face some potential limitations and challenges, including: (1) Stability and lifetime issues. 2D materials may be affected by environmental factors and chemical reactions during long-term use, resulting in a decrease in sensor performance. Future research needs to address these stability and lifetime issues to ensure long-term stable and reliable operation of the sensor; (2) size regulation and consistency. Preparing large-scale, consistent 2D materials is crucial for sensor fabrication. Although some progress has been made, the need for different sizes and shapes in different application fields still requires further research.

## Figures and Tables

**Table 1:** The Refractive index of each layer in the proposed GWSPR sensor at the wavelength of λ = 633 nm.

Materials	BK7	Au	2S2G	MoS_2_	BlueP/MoS_2_	Sensing Medium
Refractive index	1.5151 [42]	0.138 + 3.620i [29]	2.358 [47]	5.08 + 1.1723i [29]	2.85 + 0.32i [39]	1.33 + ∆*n_s_*

**Table 2:** A comparison of the results of the proposed work with previous reports.

SPR Sensors	Wavelength	Sensitivity	Ref.
Ag+ BlueP/MoS_2_	662 nm	355°/RIU	[50]
Au + BP	633 nm	245°/RIU	[58]
Au + BP + graphene	633 nm	218°/RIU	[56]
Au + BP + WSe_2_	633 nm	279°/RIU	[29]
Au + BP + MXene + Si	633 nm	264°/RIU	[59]
Ag/TiSi_2_/graphene	633 nm	183.4°/RIU	[60]
Au + Si + WS_2_	633 nm	147.88°/RIU	[61]
Ag + PtSe_2_ + graphene	633 nm	235°/RIU	[57]
Ag + Au + PtSe_2_	633 mm	165°/RIU	[62]
Au + 2S2G + blueP/MoS_2_	633 mm	290°/RIU	Our work

## Data Availability

The original contributions presented in the study are included in the article, further inquiries can be directed to the corresponding author.
